# Artificial Intelligence Integration in U.S. Healthcare Professional Degree Programs: A Rapid Scoping Review

**DOI:** 10.21203/rs.3.rs-9819693/v1

**Published:** 2026-06-19

**Authors:** Reza Taheri, Max Foroughi, Keykavous Parang

**Affiliations:** Chapman University School of Pharmacy; Chapman University School of Pharmacy; Chapman University School of Pharmacy

**Keywords:** Artificial intelligence, Health professions education, Curriculum development, Competency-based education, Scoping review

## Abstract

**Purpose:**

Artificial intelligence (AI) capabilities and use in health care have expanded rapidly, creating an urgent need to adapt health professions curricula. This study analyzed peer-reviewed evidence (2019–2026) on AI integration in health professional degree education relevant to U.S. programs, characterized study designs and findings, identified research gaps, and provided future directions for scalable, ethically governed AI curricula.

**Method:**

A rapid scoping review approach was applied using PubMed and Google Scholar as primary sources, supplemented by citation chaining of high-yield syntheses and targeted retrieval of authoritative U.S. curricular landscape resources and selected institutional exemplars (not counted as peer-reviewed studies). Inclusion targeted English-language, peer-reviewed studies (2019–2026) addressing artificial intelligence/machine learning/generative artificial intelligence education in health professions education, including surveys, curriculum evaluations, qualitative studies, consensus/Delphi, and scoping/systematic reviews. Data were extracted into evidence-mapping Tables (study characteristics and methods summary) and descriptively synthesized by profession, content domains, and evaluation outcomes.

**Results:**

Thirty-six peer-reviewed papers meeting the inclusion criteria were synthesized. Study designs were skewed toward reviews (integrative/scoping/systematic) and surveys; fewer evaluated educational interventions with objective pre/post outcomes. Medical education, especially radiology, accounted for many implementations and studies, while dentistry, pharmacy, and physical therapy education had smaller but growing bodies of work. Evidence syntheses consistently call for competency-based curricular design, faculty development, assessment redesign for generative AI, and governance aligned with ethical and risk-management frameworks.

**Conclusions:**

U.S. health professions programs are moving from ad hoc AI exposure to structured offerings emerging from national organizations and institutional exemplars. However, the literature base remains early, with limited multi-institutional evaluations, minimal longitudinal follow-up, and weak linkage to clinical performance or patient outcomes. Next-generation research should prioritize competency-linked curricula, transparent evaluation methods, and equity- and safety-centered governance.

## Teaser text

This rapid scoping review maps peer-reviewed evidence and the United States institutional landscape for the integration of artificial intelligence across 10 health professional degree programs. The authors describe what programs are teaching, where evaluation evidence is strongest and weakest, and what governance, competency, and assessment work is needed for safe, equitable, ethically grounded curricula.

## Introduction

Artificial intelligence (AI) is reshaping health care. From diagnostic imaging and clinical decision support to ambient documentation and the generative AI (GenAI) tools now used daily by learners, AI has become a defining feature of the practice environment that today’s health professions students will enter. Yet the speed of clinical AI adoption has consistently outpaced the educational frameworks needed to prepare learners to use these tools safely, effectively, and ethically. This gap is no longer a theoretical concern. It is an immediate workforce-readiness problem that every U.S. health profession program must now address.

Responses to this challenge are accelerating but remain fragmented. National bodies, including the Association of American Medical Colleges (AAMC) and the American Medical Association (AMA), have issued competency frameworks and guiding principles. Individual institutions have introduced AI courses, certificates, and, in some cases, mandatory longitudinal curricula. Scoping and systematic reviews have begun to map learner attitudes, educational interventions, and ethical considerations. Despite this activity, three gaps persist. First, the published evidence is uneven across professions, with medicine and radiology well represented while dentistry, pharmacy, nursing, physical therapy, and allied health have smaller bodies of work. Second, most evaluations focus on learner perception or short-term knowledge rather than clinical performance or patient outcomes. Third, the institutional landscape of what U.S. programs actually do is poorly captured in the peer-reviewed literature, which lags behind curricular practice.

This rapid scoping review was designed to address these three gaps in a single integrated synthesis. Prior reviews have generally examined AI within a single profession or content area. To our knowledge, none has combined a peer-reviewed evidence synthesis with a structured institutional landscape scan across ten U.S. health professional degree disciplines. By pairing these two lenses, the review describes not only what the evidence shows but also what programs are actually implementing, where gaps between evidence and practice appear, and how national guidance is shaping local curricular change.

Our objectives are therefore to (1) characterize peer-reviewed evidence on AI integration in U.S. health professional degree education across study designs and professions; (2) map the institutional landscape of curricular adoption across ten disciplines (MD (Doctor of Medicine)/DO (Doctor of Osteopathic Medicine), physician assistant (PA), pharmacy, nursing, physical therapy (PT), occupational therapy, dentistry, optometry, chiropractic, and cross-disciplinary offerings); and (3) identify research, governance, and curriculum-design priorities for scalable, equitable, and ethically governed AI education. The review is written for educators, program leaders, accreditors, and policymakers making curricular decisions now, in a field where published evidence lags implementation and where today’s choices will shape the AI competence of the next generation of clinicians.

## Methods

We used a rapid scoping review approach to map and synthesize peer-reviewed evidence on AI integration in health professional degree education relevant to U.S. programs, while separately incorporating authoritative U.S. landscape sources and institutional exemplars for contextual triangulation (not counted as peer-reviewed studies). We informed our methodology with established guidance on scoping-study design and reporting.^1,2^

### Literature identification and selection.

We conducted the primary literature search in PubMed and Google Scholar, selecting these sources for their relevance to biomedical and health professions education literature and their accessibility for rapid evidence mapping. We supplemented identification using backward and forward citation chaining from high-yield reviews, consensus papers, and profession-specific implementation studies, alongside targeted retrieval of authoritative U.S. curricular landscape sources (e.g., from the AAMC^3^) and selected institutional exemplar pages for contextual triangulation; these web-based sources were used only to characterize the U.S. implementation landscape and were not counted as peer-reviewed papers. The search window spanned January 1, 2019, through March 14, 2026. Only English-language sources were included.

### Search concepts and keywords.

Because this study was designed as a rapid scoping review rather than a fully reproducible systematic review, exact database search strings were not preserved prospectively (this limitation is explicitly acknowledged). Instead, the search followed a concept-based, iterative strategy combining AI terms (artificial intelligence, machine learning, deep learning, generative AI, large language model, LLM, ChatGPT, foundation model, multimodal, clinical decision support) and education/curriculum terms (medical education, health professions education, curriculum, undergraduate and graduate medical education, residency, nursing education, pharmacy education, dental education, PA education, PT education, competencies, Delphi, scoping review, systematic review, survey, evaluation, assessment, academic integrity) plus profession filters where relevant. Titles and abstracts were screened for relevance, and full texts were reviewed when abstracts indicated a direct curricular, educational, evaluative, or framework-related contribution.

### Inclusion and exclusion criteria.

Peer-reviewed publications in English (2019–2026) addressing AI/ML/GenAI/LLM education in health professional degree training (medicine, nursing, pharmacy, dentistry, PA, PT, allied health) or closely linked curriculum/evaluation frameworks were included. Eligible study types included scoping/systematic reviews, Delphi/consensus, surveys, curriculum implementation/evaluation studies, qualitative studies, and specialty curricula papers. Excluded were non-English publications; papers focused solely on AI clinical performance without an education integration component; and institutional web pages or policy documents (included for context only). Because exact Boolean search strings and retrieved hit counts were not retained, this manuscript functions as a rapid scoping synthesis rather than a fully reproducible systematic review.

### Data extraction and synthesis.

For each included study, we extracted the following when available: country/setting, learner population, profession, study design, AI content focus, delivery modality, and reported outcomes. We synthesized data descriptively to map the evidence base across professions and study types, and thematically to identify recurring cross-cutting domains, including competencies, pedagogy, assessment, governance, and equity. Consistent with the rapid scoping design, we aimed to characterize patterns, implementation approaches, and research gaps rather than to estimate pooled effects or perform formal quality-weighted comparisons.

## Results

### Evidence map and study design distribution

**Figure 1:**
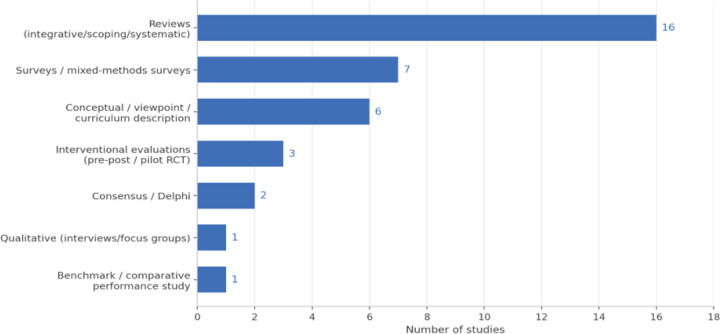
Horizontal bar chart showing the distribution of 36 included peer-reviewed studies by primary study design. Scoping/systematic/integrative reviews and cross-sectional surveys account for the majority; fewer studies are pre/post intervention evaluations, conceptual frameworks, or benchmarking comparisons.

Across the 36 included peer-reviewed papers, we found that the most common study designs were scoping, systematic, or integrative reviews and survey-based studies, whereas fewer publications evaluated educational interventions with objective pre/post outcomes (Fig. 1). Medical education, particularly radiology-focused education, accounted for a substantial share of identified implementations and published studies. We identified smaller but growing bodies of literature in dentistry, pharmacy, PA, nursing, and PT.

Across professions, we identified four recurring content domains: (1) AI fundamentals and terminology; (2) clinical applications, limitations, and critical appraisal; (3) ethics, law, policy, and bias; and (4) practical tool use under supervision. However, the evaluation spine is less mature: objective outcomes are most visible in tightly scoped specialty pilots (e.g., radiology certificate pre/post gains) and are uncommon in multi-institution implementations, a limitation highlighted in a systematic synthesis of AI educational outcomes.^4^ These findings are consistent with prior evidence syntheses reporting heterogeneous study designs, limited objective outcomes, and little evidence of higher-level behavioral or workplace effects in AI-related health professions education.

### U.S. institutional landscape across ten disciplines

**Figure 2:**
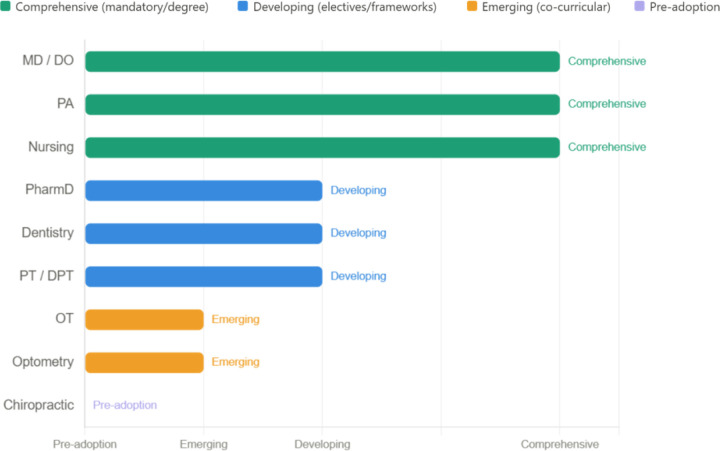
Grid summarizing artificial intelligence integration maturity across ten United States health professions disciplines. Medical (MD/DO), physician assistant, and nursing programs are shown at a Comprehensive level; pharmacy, PT/doctor of physical therapy (DPT), and dentistry at Developing; occupational therapy and optometry at Emerging; and chiropractic at Pre-adoption.

Although institutional web sources are not considered peer-reviewed evidence, they contextualize the accelerating pace of implementation in the United States. Across 10 healthcare disciplines, AI curriculum integration in U.S. professional degree programs ranges from comprehensive, mandatory frameworks to complete pre-adoption, reflecting a field in an uneven but accelerating transition (Fig. 2). Table 1 presents the institutional landscape map, a snapshot of AI curriculum integration across ten U.S. healthcare professional degree programs.

Table 2 details the study-level characteristics of all 36 included peer-reviewed papers, organized by reference number and capturing country or region, learner population and profession, study design, AI content focus, and key reported outcomes; it serves as the primary evidence map referenced throughout the synthesis that follows. Across the included literature, several recurring methodological patterns were identified. Scoping reviews commonly followed PRISMA-ScR–aligned screening approaches and relied on narrative synthesis in the context of substantial heterogeneity.^18, 34, 40, 44^ Systematic reviews of educational outcomes typically employed multi-database search strategies but yielded a limited number of eligible studies, with relatively few assessing higher-level outcomes.^4, 33^ Survey-based needs assessments were frequently cross-sectional or mixed-methods in design, incorporating Likert-scale instruments alongside open-ended responses, and often reported low-to-moderate response rates and self-identified training gaps.^17, 33, 35, 47^ Intervention evaluations most often used pre–post testing and learner satisfaction measures, with pilot or feasibility designs and inconsistent reporting of underlying educational theory or learning objectives.^20, 21, 31^ Benchmarking studies compared large language model performance with that of learners or standardized examinations, with findings sensitive to item format, particularly for media-based questions.^38^ Finally, framework and governance papers focused on proposing conceptual models and safeguards related to trust, ethics, and risk in educational implementation.^32, 42, 45^

Medical, PA, and nursing programs occupy the most advanced tier, with institutions such as Stanford Medicine, Harvard, and Florida State University embedding AI as a required component of professional formation, supported by dedicated degree pathways, clinical practicum hours, and institutional tool infrastructure. Stanford Medicine’s mandatory AI curriculum (effective Fall 2025) applies to all MD and PA students and is structured around four competencies: foundational knowledge, clinical integration skills, ethical/legal frameworks, and critical model appraisal.^5^ Harvard’s HST track mandates a two-course AI sequence (HT16 and HT18) and runs a parallel PhD track in AI in Medicine.^6^ UT Health San Antonio offers an MD/AI dual degree and longitudinal electives, including ELEC 5070 and ELEC 5080.^7^ According to the 2024 AAMC/AACOM Curriculum SCOPE Survey (88% response rate across 208 MD- and DO-granting schools), 77% of responding institutions had incorporated AI into their curricula, a 59% increase from 53% in 2023.^8^ Of those schools, 107 confirmed AI was in the required curriculum. Notably, only 47% of surveyed schools had an appropriate AI use policy, and just 30% provided learners with secure access to an AI agent, indicating that governance infrastructure is lagging significantly behind curricular adoption.

Nursing has produced the field’s most innovative credential. For example, Florida State University’s MSN with AI Concentration, the first of its kind in the U.S., requires 500 hours of integrated coursework and clinical application and prepares graduates for roles such as Chief Clinical AI Officer.

Pharmacy programs are actively developing their approaches, with dual-degree offerings and multi-course sequences emerging at leading schools, though they have yet to meet universal accreditation mandates. No accreditation body mandates AI competencies across PharmD programs as of January 2026, though the American Society of Health-System Pharmacists (ASHP) and the American Association of Colleges of Pharmacy (AACP) are actively developing frameworks. Long Island University’s PharmD/MS in AI dual degree and the University of Florida’s multi-course AI curriculum represent the leading edge.

Allied health disciplines (PT, occupational therapy, dentistry, optometry) show growing but unsystematic adoption, largely through tool integration and faculty-driven co-curricular initiatives rather than required curriculum. PT and occupational therapy show early-stage integration, largely through faculty-driven tool adoption and co-curricular training rather than formalized curriculum requirements. Optometry sits at a similar stage, with AI finding its way into imaging and diagnostics instruction at select institutions. Chiropractic education remains a notable gap: professional bodies acknowledge AI’s relevance, yet no program with a formal curriculum requirement was identified as of December 2025.

Cross-disciplinary national resources partially bridge these gaps. The AAMC has produced the most developed body of organized guidance for U.S. health professions education. Its Artificial Intelligence and Academic Medicine initiative provides a coordinated collection of resources spanning policy principles, competency frameworks, and practical implementation tools.^3^ The AAMC’s AI Competencies Across the Learning Continuum articulate progressive expectations for learners from undergraduate medical education through continuing professional development, organized around domains that include foundational knowledge, clinical decision-making, ethics, and communication.^9^ Complementing the competencies, the AAMC’s Principles for AI Use, maintaining a human-centered focus, ensuring ethical and transparent use, providing equal access, fostering education, and developing curricula, offer institutions a shared values framework for AI governance and curricular design.^10–14^ Together, these documents supply durable scaffolding that single-discipline curricula have so far lacked, and they are becoming reference points for programs drafting local policies and required learning outcomes. What remains missing is an equivalent body of guidance from accreditors and professional societies outside medicine.

Taken together, the pattern suggests that AI integration is being driven less by top-down accreditation mandates than by institutional leadership and faculty initiative, underscoring both the opportunity and the urgency for standardized competency frameworks across the health professions. The interprofessional dimension remains underemphasized across all disciplines: because AI-enabled clinical teams will include physicians, nurses, pharmacists, dentists, PAs, and PTs collaborating around shared decision-support outputs, siloed profession-specific AI curricula risk creating AI literacy gaps that undermine team-based care. Several competency frameworks explicitly call for interprofessional AI education, yet the peerreviewed literature contains few examples of such implementation.^15^

Qualitative work on AI-enhanced blended learning further shows how curricular design can reshape learner behaviors and perceived competence, while also revealing concerns about reliance, accuracy, and alignment with educational goals.^16^ Institutional exemplars identified through web-based scanning. Modular AI curricula, dedicated AI coursework in health care tracks, and institution-wide deployment of secure GenAI platforms for students illustrate a rapidly evolving practice that is often ahead of the peer-reviewed evaluation literature.

## Discussion

### Cross-disciplinary observations

The peer-reviewed literature indicates that U.S. AI integration in health professional education is transitioning from “why AI matters” to “how to teach and govern AI use,” but the transition is uneven across professions and is constrained by faculty capacity, curricular crowding, and evaluation limitations. A recent scoping review of generative AI guidelines across 21 universities in 15 countries found no dental education-specific guidance documents, underscoring the governance gap in this discipline.^43^ The included literature was dominated by reviews, surveys, and conceptual or framework-oriented papers, whereas fewer studies reported primary evaluations of educational interventions or higher-level outcomes. Consensus-oriented frameworks explicitly caution about curriculum space and recommend focusing on durable foundational concepts rather than transient technical details, a strategy that also aligns with risk-management logic and the need for learner critical appraisal skills.^25^

Across disciplines, surveys consistently show high learner and faculty interest coupled with low formal training penetration and concerns about ethics and academic integrity. In U.S. medical student data, respondents expressed a desire for AI training while reporting limited school resources;^46^ in PA education, faculty reported positive orientations but low confidence without training, with concerns centering on critical thinking and integrity.^17^ Nursing students and nursing-education syntheses similarly emphasize benefits but highlight reliance, accuracy, and support needs, suggesting that “AI literacy” must be operationalized with explicit instruction in verification, uncertainty, and professional accountability.

While limited, the evidence shows that structured curricula can produce measurable gains, particularly when educational objectives are concrete and theory-informed. The radiology AI certificate program provides the clearest U.S. example of a pre/post evaluation with objective knowledge metrics, showing mean score improvement from 37% to 73%.^31^ A pilot randomized controlled trial of LLM-based clinical reasoning training in PT showed feasibility constraints, such as high recruitment but low active engagement, suggesting that sustained design and duration are critical parameters.^21^

Several structural observations emerge from cross-disciplinary comparison. First, medical and PA programs benefit from early accreditation engagement (e.g., AAMC competency frameworks)^9^ and institutional AI infrastructure investment that other disciplines lack. Second, nursing’s FSU MSN-AI track demonstrates that a dedicated graduate credential is achievable and market-responsive, offering a model for other disciplines. Third, pharmacy’s situation that is characterized by dual-degree innovation at the margins and regulatory absence at the center, echoes earlier patterns in evidence-based medicine adoption, where professional society leadership preceded accreditation mandates. Fourth, the complete absence of formal AI curriculum in chiropractic education, despite clinical AI’s growing relevance in imaging and practice management, represents a professional organization leadership opportunity.

### Dentistry: a case for profession-specific curriculum development

Dentistry deserves separate treatment within this review because its educational use cases are unusually concrete and clinically proximate. Existing dental literature shows that AI is being applied not only to didactic support but also to diagnostic training, image interpretation, simulation, assessment, and learner feedback, while recent GenAI-focused commentaries and scoping reviews highlight the rapid expansion of LLM and multimodal foundation-model use across teaching workflows.^22,28^ At the same time, dental education remains underdeveloped compared with medicine: published work is dominated by perspectives and scoping reviews, standardized competency frameworks are lacking, and guidance for educators on responsible implementation is still emerging. This imbalance is especially important in dentistry because many near-term applications, such as radiology decision support, caries detection, orthodontic analysis, and charting assistance, sit close to real patient care and can shape clinical reasoning early in training.^23, 49–52^

Dentistry also presents a distinctive governance challenge because AI is entering both the educational and clinical environments through the same high-visibility diagnostic workflows, especially radiographic interpretation, caries detection, orthodontic planning, and documentation support. That overlap increases the importance of teaching verification, calibration, supervision, and medico-legal accountability rather than framing AI as a purely technical add-on. In practical terms, dental curricula should require learners to compare AI outputs with faculty judgment and reference standards, document disagreement rationales, and recognize when model outputs are unreliable because of image quality, population mismatch, or limited task scope.^22, 43, 53–55^

A dentistry-specific curricular model should therefore move beyond generic “AI awareness” and organize training around four applied domains: (1) imaging and diagnostic support, including interpretation of radiographs, CBCT, photographs, and intraoral scans; (2) treatment-planning augmentation, including triage, risk stratification, and restorative/orthodontic workflow support; (3) generative-AI use in documentation, patient communication, and learning support; and (4) ethics, privacy, bias, and regulatory boundaries for tools used in educational and clinical settings. For dental programs, the most defensible early assessments are structured image-appraisal exercises, AI-assisted case write-ups with mandatory source verification, OSCE-style stations using simulated AI outputs, and reflective audits of when students appropriately reject or override an AI recommendation.^22, 23, 28, 55, 56^

### Critical analysis and research gaps

The most persistent gap is the mismatch between the speed of institutional adoption and the maturity of evaluation science for AI-enabled education. A systematic review of AI educational outcomes found limited rigor, weak reporting of learning objectives, poor control for confounding factors, and outcomes concentrated at lower evaluation levels, with minimal evidence of workplace behavior or patient care effects.^4^ This limitation is amplified by the proprietary and rapidly evolving nature of many AI systems (including GenAI), which complicates reproducibility and long-term curricular planning.

Profession-specific gaps remain pronounced. Medicine, especially radiology, has more structured curricular work and published evaluations, whereas the pharmacy education literature emphasizes academic-integrity risks and a paucity of peer-reviewed mitigation strategies. Dentistry and nursing education have growing scoping/perspective literature, but fewer controlled intervention studies. For PT and allied health, a small number of newer studies exist (course evaluations, feasibility trials, tool acceptance research), but systematic, multi-site outcomes research remains limited. In endodontics specifically, a scoping review identified ten domains where AI could support education, from radiographic interpretation to autonomous systems, yet none had been validated through controlled educational trials.^23^

Governance and equity gaps are frequently acknowledged but under-measured. Ethics-focused scoping work documents recurring concerns (privacy, bias, transparency, consent), but the field lacks standardized, measurable educational outcomes for “ethical AI practice” and robust evaluations that track differential impacts on learners and populations.^34^ These issues intersect with national and international guidance that emphasizes trustworthy AI risk management and ethical governance, suggesting that curricula should include not only technical literacy but also operational governance competencies.

A cross-cutting methodological limitation deserves explicit note: the majority of studies reviewed here measure learner perception or short-term knowledge (Kirkpatrick levels 1–2) rather than behavioral change in clinical settings or patient outcomes (levels 3–4). This reflects a structural challenge of AI education research, the difficulty of isolating curriculum effects in dynamic clinical environments with rapidly evolving tools. Future work should consider quasi-experimental designs, electronic health record-linked outcome metrics, and simulation-based behavioral assessments as intermediate markers.

### Conceptual framework for integrating evidence

**Figure 3:**
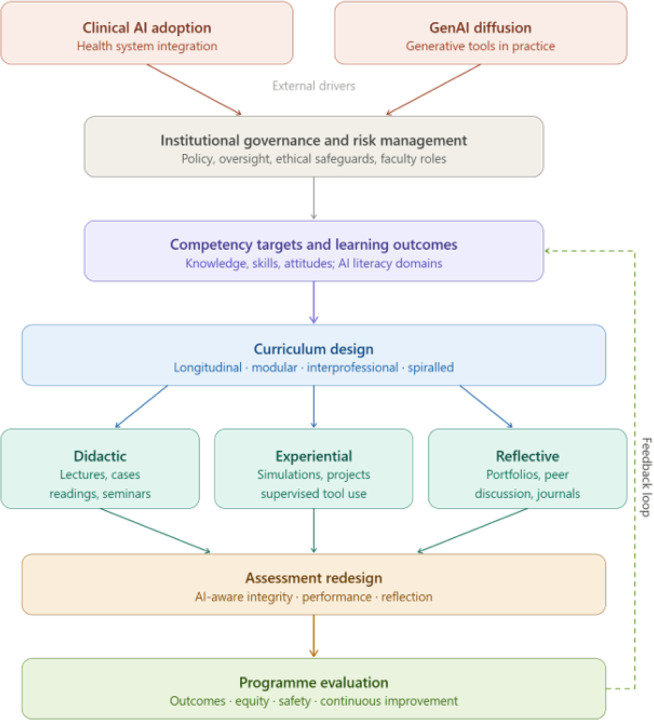
Top-down logic model with seven sequential tiers. External drivers (clinical artificial intelligence adoption and generative artificial intelligence diffusion) flow into institutional governance and risk management, then competency targets, curriculum design, three teaching modalities (didactic, experiential, reflective), assessment redesign, and program evaluation. A dashed feedback arrow returns evaluation findings to the competency layer.

To contextualize the findings above and provide a synthesis lens for the discussion that follows, Fig. 3 presents a framework for integrating AI into health professions curricula, organized as a top-down logic model with seven sequential tiers connected by a continuous feedback loop. At the apex, two external drivers, clinical AI adoption and GenAI diffusion, represent the real-world pressures compelling educational institutions to act. These forces converge downward into institutional governance and risk management, the structural layer that translates external demand into institutional policy, ethical oversight, and defined faculty responsibilities. Governance, in turn, informs the specification of competency targets and learning outcomes, where the curriculum’s intellectual ambitions are articulated across knowledge, skills, attitudes, and AI literacy domains.

From competencies, the framework descends into curriculum design proper, conceived as longitudinal, modular, interprofessional, and spiraled, ensuring AI content is not siloed in a single course but woven progressively across the full program. Curriculum design then branches into three complementary teaching modalities: didactic instruction (lectures, cases, readings, seminars), experiential learning (simulations, projects, supervised tool use), and reflective practice (portfolios, peer discussion, journals). This tripartite delivery structure acknowledges that AI competency requires not only conceptual understanding but also hands-on application and critical self-examination.

All three modalities feed into assessment redesign, which must respond to the AI context through integrity frameworks, performance measurement, and reflective evaluation. Assessment outcomes then flow into program evaluation, the framework’s terminal layer, which monitors outcomes, equity, safety, and opportunities for continuous improvement. A dashed feedback arrow returns evaluation evidence directly to the competency layer, closing the loop and ensuring the framework is self-correcting. This architecture positions AI curriculum integration not as a one-time reform but as an iterative, evidence-driven process responsive to both institutional realities and evolving clinical practice. This model aligns with recent proposals for structured GenAI implementation frameworks in health education, which similarly emphasize student AI literacy, educator capability, and assessment redesign as three core pillars.^42^

### Future Directions

Future research and curriculum development for U.S. health professional degree programs should prioritize five convergent directions supported by the reviewed evidence and authoritative guidance. This need is especially acute in dentistry, where clinically adjacent AI applications are expanding faster than profession-specific curricular standards. A recent review confirmed that no dental education-specific GenAI guidelines currently exist among surveyed institutions internationally, making this gap both documented and actionable.^43^

First, curricula should be explicitly competency-based and aligned with emerging national competency efforts, ensuring that “AI literacy” includes critical appraisal, bias recognition, limitations, and accountable use rather than tool familiarity alone.

Second, evaluation designs should move beyond short-term attitudes and isolated knowledge tests and toward multi-institutional, theory-informed trials that measure authentic performance (e.g., clinical reasoning tasks, documentation quality, evidence synthesis, communication) with longitudinal follow-up. The methodological shortcomings documented in outcomes syntheses should be treated as a research agenda rather than a footnote.

Third, assessment and academic integrity must be redesigned for GenAI realities, especially in pharmacy, nursing, PA, and medicine, where survey and scoping evidence repeatedly flags integrity and overreliance concerns. This includes explicit instruction on verification practices and responsible tool use, plus assessments that evaluate reasoning and process rather than only product.

Fourth, institutional governance should be embedded into curricula and faculty development, using recognized risk and ethics guidance from international and U.S. standards bodies. In practice, this means teaching learners how AI systems are governed (data governance, privacy, auditability, transparency) and how clinicians participate in safe deployment.

Fifth, interprofessional curriculum models should be expanded and evaluated. Given that AI-enabled care is inherently team-based and spans clinical, informatics, regulatory, and operational domains, cross-profession curricula (medicine/DO, nursing, pharmacy, dentistry, PA, PT, allied health) may improve efficiency and shared mental models, but require tailored professional contexts and profession-specific competencies. Evidence mapping suggests that current literature remains siloed by discipline and specialty; interprofessional research could close this gap.

## Conclusions

Evidence published between January 1, 2019 and March 14, 2026 indicates rapidly rising interest and early-stage implementation of AI, including GenAI and LLMs, across U.S. health professions education. Peer-reviewed literature remains dominated by scoping/systematic reviews, surveys, and conceptual frameworks, with comparatively few rigorously evaluated curricular interventions demonstrating higher-level learning, clinical behavior change, or patient outcomes. This asymmetry, high implementation pressure with low evidence of outcomes, is repeatedly highlighted in analyses of AI-enabled education outcomes and is amplified by GenAI’s diffusion into learner workflows, assessments, and institutional policy discussions. Closing this gap will require competency-linked curricula, transparent and multi-institutional evaluation, and governance anchored in equity and safety.

## Figures and Tables

**Table 1:** Artificial intelligence integration in U.S. healthcare professional degree programs (institutional landscape).

Discipline	Institution(s)	Key program / offering	Integration level	Notable feature
MD/DO	Stanford, Harvard, UT Health SA, GWU, UVA, Mt Sinai, PCOM, KansasCOM	Mandatory AI curriculum (Stanford, Fall 2025)^5^; AI in Medicine PhD track (Harvard)^6^; MD/AI dual degree (UT Health SA)^7^	Comprehensive	Stanford’s 4-competency framework is mandatory for all MD + Pa students; Harvard HST requires HT16 & HT18; ~77% of U.S. MD/DO-granting institutions have incorporated AI in some form^8,9^
PA	Stanford, Univ. of Pittsburgh, Univ. of Lynchburg^a^	Shared mandatory AI curriculum with MD (Stanford); Digital Health Fundamentals (Pitt DMSc); AI in Healthcare certificate (Lynchburg)	Comprehensive	Lynchburg’s 10-credit AI certificate earns up to 60 AAPA Category 1 CME credits; faculty surveys show positive orientation but low confidence without training^17^
PharmD	LIU, UF, USC Mann, Purdue, ASHP, AACP^a^	PharmD/MS in AI dual degree (LIU, 5 yrs); multi-course AI curriculum (UF); Digital Health & AI elective (USC, PHRD 599)	Developing	No universal ACPE AI mandate yet; ASHP & AACP actively building frameworks as of 2025–26; scoping work documents academic-integrity risks and limited peer-reviewed mitigation strategies^18^
Nursing	Florida State Univ. (FSU), Univ. of Florida, Northern Illinois Univ.^a^	First-of-its-kind MSN with AI Concentration (FSU, 500 clinical hours); AI competency framework (UF)	Comprehensive	FSU prepares nurses for Chief Clinical AI Officer roles; fully asynchronous online delivery; scoping reviews highlight implementation challenges and need for continuous evaluation^19^
PT/DPT	Univ. of Pittsburgh (pitt), Hawaii Pacific University (HPU)^a^	AI anatomy chatbot in DPT core (Pitt); AI tools in Evidence-Based Practice II (HPU)	Developing	HPU study showed significant improvement in AI literacy (p < 0.05) and research self-efficacy^20^; pilot RCT of LLM-based clinical reasoning training shows feasibility constraints^21^
OT	Boston University (BU),^a^ Korro AI	Grant-funded online AI training for OT/allied health students and faculty (BU); Korro AI tool integration	Emerging	No formal curricular AI components identified; training is co-curricular and faculty-driven.
Dentistry	Univ. of Florida (UF), UCLA^a^	AI across the curriculum via $70M NVIDIA partnership (UF); AI in dental diagnostics and imaging (UCLA)	Developing	UF designated as nation’s first ‘AI University’; scoping work identifies lack of standardized frameworks and clinician-led development as key gaps^22^
Optometry	Midwestern Univ. Arizona^a^	AI integration in ophthalmic diagnostics and imaging curricula	Emerging	Clinical imaging AI (e.g., diabetic retinopathy screening) is the main application domain; single institution profiled.
Chiropractic	Not identified	No programs with formal AI curriculum requirements identified as of December 2025	Pre-adoption	Professional support for AI exists, but formal curriculum integration is absent; this represents a significant opportunity for professional organization leadership.
Cross-disciplinary	UIUC, Univ. of Lynchburg,^a^ AMA Ed Hub	AMA CME course ’Practical Applications for AI in Health Care’ (23.25 credits); AI in Healthcare certificate (Lynchburg)	Developing	AMA CME course available to all health professionals; active and updated as of early 2026; cross-disciplinary resources partially bridge discipline-specific gaps.^9–14^

a Source: Verified via institutional websites and official program announcements.

**Table 2:** Study characteristics and key findings (peer-reviewed evidence synthesis; n = 36).

Reference	Country/region	Learner population/profession	Design	AI content focus	Key outcomes/findings (as reported)
4	International	Health professions education	Systematic review	Educational outcomes of AI-based training/assessment	Included 12 studies; evidence quality poor, mostly Kirkpatrick level 2; limited reporting of learning objectives and confounding control; called for rigorous methods.
8	United States/Canada	MD/DO schools (AAMC/AACOM SCOPE survey)	National curriculum survey data snapshot	AI curriculum adoption, required status, and governance policies	77% of responding schools had incorporated AI; 107 schools had AI in the required curriculum; only 47% had an AI use policy, and 30% provided secure AI agent access.
15	International	Clinical AI integration teams	Conceptual/review	Scientist–clinician collaboration in clinical AI	Highlighted collaboration gaps relevant to interprofessional AI education.
16	Germany	Medical students	Qualitative interview study	AI-driven blended learning experiences	Reported learner perceptions of AI-supported blended learning; emphasized both perceived learning benefits and concerns requiring instructional design and oversight.
17	Multi-country (US PA programs)	PA/physician associate faculty	Survey (Likert + open comments)	Faculty perceptions of AI in PA education; training needs; academic integrity	Most respondents expressed positive views; concerns about critical thinking and integrity; participation in AI-related continuing education was associated with higher confidence.
18	Australia	Pharmacy education literature	Scoping review	GenAI use and academic integrity	Identified published (n = 12) and gray (n = 9) records; noted paucity of peer-reviewed mitigation strategies.
19	International	Nursing education (hospital settings)	Scoping review	AI implementation in nursing education framed via SWOT	Selected 15 studies from 6,517; identified positives and challenges (technical issues, language barriers, limited realism).
20	United States	Doctor of Physical Therapy (DPT) students	Course evaluation (pre/post)	AI integration in the evidence-based practice course	Measured changes in selfefficacy and AI literacy metrics.
21	Spain	PT students	Pilot randomized parallel-group study (feasibility)	LLM (GPT-4) for clinical reasoning case training	High recruitment/participation but low active engagement; feasibility constraints identified.
22	Australia	Dental student education	Scoping review	AI applications for learning, assessment, and diagnostic training	Identified 17 included studies from 547; categorized domains; highlighted the lack of standardized frameworks and clinician-led development.
23	International	Endodontics education	Scoping review	AI applications in endodontics education	Identified ten domains where AI could support endodontic education, from radiographic interpretation to autonomous systems; none validated through controlled educational trials.
24	International	Medical education stakeholders	e-Delphi	AI competencies and curricular domains	Produced consensus-based competency recommendations for AI in medical education.
25	International	Medical education/digital health expert panel	Consensus (modified Delphi)	Digital health competencies framework (AI-relevant within digital health domains)	Developed DECODE framework with domains/competencies and learning outcomes; highlighted curriculum space/implementation challenges; recommended foundational concepts over rapidly changing technical details.
26	International	Medical education literature	Integrative review	Applications and challenges of AI in medical education	Synthesized applications/challenges; emphasized implementation complexities and curricular implications.
27	International	Health care professionals across training stages	Scoping review	AI education programs and recommended content	Identified 41 relevant studies from > 10,000 citations; distinguished described programs vs. recommended content; categorized cognitive/psychomotor/affective domains.
28	United States	Dental education (viewpoint)	Viewpoint	LLMs and multimodal foundation models in dental education	Described opportunities (personalized feedback, scenarios, content generation) and risks (bias, inaccuracy, privacy, overreliance).
29	International	Medical imaging staff/learners	Scoping review	AI educational programs in medical imaging	Mapped AI educational program content/delivery for imaging workforce; highlighted implementation considerations.
30	Egypt/UAE	Undergraduate PT students	Comparative cross-sectional survey	ChatGPT usefulness/ease-of-use; usage patterns	Users reported higher perceived usefulness/ease-of-use; usage focused on theoretical/simple cognitive tasks; concluded the need for structured AI training in PT education.
31	United States	Radiology residents	Prospective pilot evaluation	AI certificate program (module-based)	Mean knowledge scores increased from 37% pre to 73% post; majority endorsed improved familiarity.
32	United States	Health professions education (conceptual)	Conceptual framework	Entrustment/EPAs for AI use in health professions education activities	Proposed AI-specific entrustment framework using trustworthiness dimensions (ability, integrity, benevolence).
33	United States (NYC sample)	Undergraduate nursing students	Survey with open-ended questions	Nursing student GenAI use and support needs	Most participants used GenAI for concept clarification and academic support; concerns about accuracy, reliance, cost.
34	International	Medical education settings	Scoping review	Ethical considerations for teaching with AI	Analyzed 82 peer-reviewed articles (2018–Oct 2023); mapped ethical themes (privacy, bias, consent, transparency).
35	United States	Medical students (MD/DO programs)	Mixed methods survey study	Student perceptions; desired AI curriculum topics/formats	Low reported formal AI training; high interest in learning; identified preferred formats/topics and perceived resource gaps.
36	International	Health professions education students	Systematic review	Generative AI use in formal training programs	GenAI most frequently applied to practice (73%), inquiry (70%), production (67%); collaborative learning modes underrepresented (12%).
37	United States	Medical education stakeholders	Scoping review	Generative AI in medical education	Mapped opportunities and challenges of GenAI tools (e.g., ChatGPT) for education; emphasized risks and future directions.
38	Germany	Medical licensing exam context	Comparative performance study	GPT-4, Bing, GPT-3.5 on German medical state exam questions	GPT-4 and Bing exceeded average medical student performance; performance varied with media-based items.
39	United States	Radiology residents	Curriculum implementation description	Integrating AI/imaging informatics as an advanced track	Described a structured residency AI/informatics training track approach within radiology training.
40	International	Medical students/residents/physicians	Scoping review	AI curriculum frameworks in medical education	Only 2 of 21 reviewed papers described actual frameworks; none guided by educational theory.
41	International	Medical education researchers/educators	AMEE Guide	Fundamentals of AI in medical education research	Provided guidance for AI-related medical education research.
42	International	Health education	Scoping review + framework	GenAI implementation framework	Proposed three pillars: student AI literacy, educator capability, and assessment redesign.
43	International	Dental education (21 universities, 15 countries)	Scoping review of institutional guidelines	GenAI guidelines in dental education	Found no dental education–specific GenAI guidance documents.
44	International (many North American studies)	Graduate medical education	Scoping review	AI/LLMs in GME perceptions, applications, practice	Included 102 studies; radiology as highest publication specialty; noted heterogeneity and evolving AI tools.
45	United States	Undergraduate medical education	Perspective	Reforming medical education for AI era	Argued that medical education should shift toward knowledge management and effective AI use.
46	United States	Medical students	Empirical study (pilot survey)	Medical student opinions on AI/LLMs in education	Reported student perceptions and concerns/anticipated uses of LLMs.
47	United States	Medical students	Empirical study (multi-institutional survey)	Medical student experiences with ChatGPT	Reported student experiences with ChatGPT in medical education, documenting usage patterns, perceived benefits, and concerns.
48	International	Nursing education literature	Scoping review	ChatGPT in nursing: opinions and educational implications	Mapped published opinions/uses and concerns about ChatGPT in nursing education. limited

AI = artificial intelligence; EPA = entrustable professional activity; GenAI = generative artificial intelligence; LLM = large language model; PA = physician assistant; PT = physical therapy

## Data Availability

Not applicable. This manuscript is a synthesis of publicly available, peer-reviewed literature and institutional web sources; no primary data were generated.
